# Intravoxel incoherent motion imaging combined with diffusion kurtosis imaging to assess the response to radiotherapy in a rabbit VX2 malignant bone tumor model

**DOI:** 10.1186/s40644-022-00488-w

**Published:** 2022-09-05

**Authors:** Jia Guo, Weikai Sun, Cheng Dong, Zengjie Wu, Xiaoli Li, Ruizhi Zhou, Wenjian Xu

**Affiliations:** 1 Department of Radiology, The Affiliated Hospital of Qingdao University, 16 Jiangsu Road, Qingdao, Shandong China; 2 Department of Radiology, Qilu Hospital, Cheeloo College of Medicine, Shandong University, Jinan, Shandong China

**Keywords:** Magnetic resonance imaging, Neoplasms, Bone, Diffusion

## Abstract

**Purpose:**

To combine intravoxel incoherent motion (IVIM) imaging and diffusion kurtosis imaging (DKI) parameters for the evaluation of radiotherapy response in rabbit VX2 malignant bone tumor model.

**Material and methods:**

Forty-seven rabbits with bone tumor were prospectively enrolled and divided into pre-treatment, considerable effect and slight effect group. Treatment response was evaluated using IVIM-DKI. IVIM-based parameters (tissue diffusion [Dt], pseudo-diffusion [Dp], perfusion fraction [fp]), and DKI-based parameters (mean diffusion coefficient [MD] and mean kurtosis [MK]) were calculated for each animal. Corresponding changes in MRI parameters before and after radiotherapy in each group were studied with one-way ANOVA. Correlations of diffusion parameters of IVIM and DKI model were computed using Pearson’s correlation test. A diagnostic model combining different diffusion parameters was established using binary logistic regression, and its ROC curve was used to evaluate its diagnostic performance for determining considerable and slight effect to malignant bone tumor.

**Results:**

After radiotherapy, Dt and MD increased, whereas fp and MK decreased (*p* <  0.05). The differences in Dt, fp, MD, and MK between considerable effect and slight effect groups were statistically significant (*p* <  0.05). A combination of Dt, fp, and MK had the best diagnostic performance for differentiating considerable effect from slight effect (AUC = 0.913, *p* <  0.001).

**Conclusions:**

A combination of IVIM- and DKI-based parameters allowed the non-invasive assessment of cellular, vascular, and microstructural changes in malignant bone tumors after radiotherapy, and holds great potential for monitoring the efficacy of tumor radiotherapy.

**Supplementary Information:**

The online version contains supplementary material available at 10.1186/s40644-022-00488-w.

## Introduction

Although primary malignant bone tumors are among the rarest types of cancer, they still pose a serious threat to human health on account of their high mortality, disability, and metastasis rates [1]. With the development of neoadjuvant chemoradiotherapy combined with surgical resection, the long-term survival rate of patients with bone malignancies has significantly increased from 20 to 70% [2, 3]. However, ineffective treatment may inevitably lead to serious side effects, such as the radiation damage or the formation of resistant clones. Accordingly, in patients with malignant bone tumor, accurate early prediction of response to neoadjuvant chemoradiotherapy is essential for determining the most appropriate treatment strategy.

Diffusion-weighted imaging (DWI) can be used to acquire apparent diffusion coefficient (ADC) values that can capture biological information such as intratumoral heterogeneity and quantify the dispersion state of water molecules within tissue, thereby providing certain advantages in response evaluation and prognosis prediction [4, 5]. However, given the complex cellular microstructural barriers within tumors, the water diffusion behavior within tumors can be complicated. Intravoxel incoherent motion (IVIM) is an advanced MRI diffusion technique that uses a double exponential model to accurately reflect the microscopic cell density and provide hemodynamic information at the molecular level through multi-b-value scanning. Previous studies showed that quantitative IVIM-derived parameters have potential value for evaluating the heterogeneity of tumor neovascularization and the tumor microvascular system [6, 7]. Diffusion kurtosis imaging (DKI), introduced by Jensen et al., can quantitively estimate the skewed distribution of water diffusion through a non-Gaussian distribution model based on a probability distribution function [8]. Recent studies applying the DKI model at ultrahigh b-values suggested that DKI-derived parameters were more sensitive to heterogeneous and irregular tumor intracellular environments than conventional monoexponential DWI, and were better for differentiating between benign and malignant bone tumors [9]. Using the parameters derived from novel DWI models, which reflect biological properties such as cellularity, vascularity, and microstructural heterogeneity, numerous investigators have evaluated multiple diffusion techniques for clinical needs such as tumor diagnosis, differentiation, and grading [10–12]. In this respect, much attention has been devoted to comparing the performance of different derived parameters with the aim of identifying the most promising diffusion model or biomarker for specific clinical purposes. For example, Xiao et al. compared the predictive effect of histogram metrics calculated from monoexponential and advanced DWI in sinonasal malignant tumors [13], and Granate et al. evaluated Gaussian and non-Gaussian DWI models for the differentiation of pancreatic lesions [14]. However, quantitative IVIM parameters may lack expression of the complexity of tissue microstructure, whereas the ultra-high b-value imaging of DKI may also be affected by perfusion. Therefore, the integration of these DWI-derived parameters could pave the way for more comprehensive tumor characterization.

Our study combines IVIM and DKI parameters for the evaluation of radiotherapy response in rabbits with VX2 malignant bone tumor model, integrating different DWI-derived biological information. In addition, correlations of IVIM and DKI-derived parameters reflecting vascularity, cellularity, and microstructural complexity were investigated. To the best of our knowledge, research on using combined IVIM and DKI to monitor therapeutic effects on skeleton malignancies is relatively limited.

## Methods

### Research objects

**Fig. 1:**
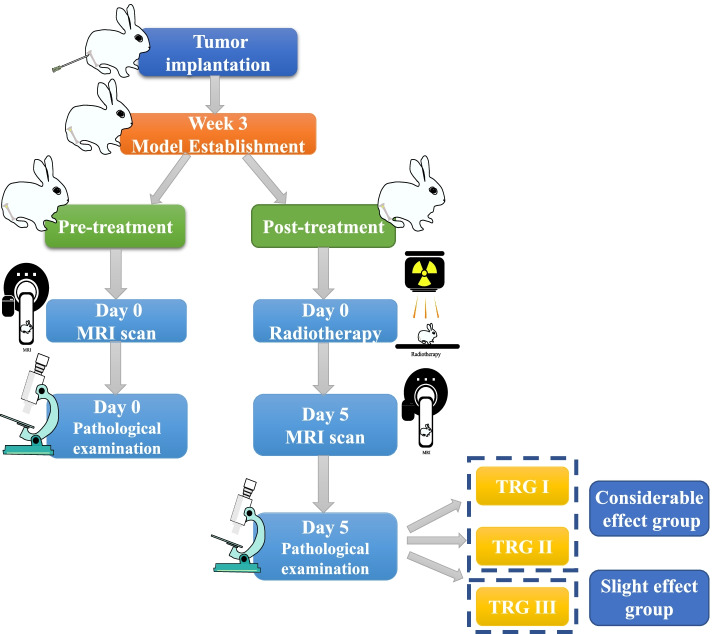
Study workflow

This study was approved by our institutional animal care committee and hospital ethics committee. Following the methods of previous studies, VX2 tumor cells (Central Laboratory of Shanghai General Hospital) and male New Zealand white rabbits (Qingdao Administration for Market Regulation) were used to establish malignant bone tumor model (*n* = 50) [15, 16]. All rabbits were 2 months of age at tumor seeding and weighed 2.0–3.0 kg. The animals were maintained in a specific pathogen-free environment with a room temperature of 22 °C–25 °C and a circadian rhythm of 12/12 light/dark. Tumor nodules were cut into 1-2 mm^3^ tissue chunks and immersed in 0.9% sodium chloride injection. For implantation, the prepared VX2 tissue fragments were delivered into the medullary cavity at the level of the right tibial nodule at a depth of 1 cm. Follow-up examinations were performed when the tumor was larger than 10 mm in diameter after 3 weeks. Considering that the same animal could not be repeated for pathological examination, and that all animals used in this study were subjected to the same survival and exposure conditions, the animals with bone tumor were divided into pre-treatment and post-treatment groups according to the principle of randomized control with a ratio of 3:7. In the pre-treatment group, baseline MRI scans were performed after model establishment. The post-treatment group underwent the intensity modulated radiation therapy (total dose 10 Gy, radiation field 6 × 6 cm^2^) and was then subjected to longitudinal MRI scanning after 5 days. Animals were sacrificed at the end of MR examination and pathological examination was performed. The post-treatment group was further divided into slight effect group and considerable effect group according to the pathological assessment. The workflow is shown in Fig. 1.

### MRI acquisition

The phantom experiment of rabbit malignant bone tumor model was tested before the MRI scanning to evaluate the accuracy and repeatability of the quantitative IVIM-DKI parameters used in this study. Details of this investigation are shown in Supplement A1. All MRI was acquired using a 3 T MRI system (Prisma, Siemens, and Erlangen, Germany) with an eight-channel rabbit coil (Chenguang, China). Ketamine (1.5 mg/kg) was injected into the thigh muscle to maintain rabbit anesthesia. The rabbits were place in a supine position and allowed to breathe freely during data acquisition. The sequences included sagittal T2-weighted fat-saturated (FS) images (echo time/repetition time, 96/3000 ms; FOV, 160 × 160 mm; matrix, 320 × 320; slice thickness, 3 mm; intersection gap, 1 mm; and number of excitations, 2) and sagittal T1-weighted FS fast spin-echo (FSE; echo time/repetition time, 22/737 ms; FOV, 160 × 160 mm; matrix, 320 × 320; slice thickness, 3 mm; intersection gap, 1 mm; and number of excitations, 2). IVIM and DKI were based on readout-segmented long variable echo-trains using the RESOLVE technique (GRAPPA, *R* = 2; echo time/repetition time, 55/3000 ms; FOV, 170 × 170 mm; matrix, 128 × 100; slice thickness, 3 mm; diffusion gradient directions, 3; number of excitations, 4; receiver bandwidth, 930 Hz/pixel; b values 0, 10, 20, 30, 40, 50, 80, 100, 150, 200, 400, 600, 800, 1000, 1500, and 2000 s/mm^2^; and scanning time: 7.4 minutes).

### Histopathology analysis

After MRI was completed, all rabbits were sacrificed and the right tibia specimens were harvested. According to the direction and thickness of the MR scan, the largest sagittal plane of the tumor was selected, and the tumor was sliced into 3-mm thick slices. The gross pathology was recorded and hematoxylin-eosin (HE) staining was performed. The pathologists evaluated the tumor cell density in the solid area by point-to-point comparison with gross slice under high- power microscope (200×). Tumor cell density was calculated as: Tumor cell density = Area_tumor cell nucleus_/ Area_statistical field_. Treatment response was assessed by classifying the degree of tumor regression into three grades based on the decrease in tumor cell density. Tumor regression of 0–40% was defined as grade I, 40–60% as grade II, and > 60% as grade III. Grade I (tumor cell regression < 40%) was defined as slight effect, while grade II or grade III (tumor cell regression > 40%) was defined as considerable effect [17]. All pathologic analyses were performed by one experienced senior pathologist with more than 5 years of experience.

### Image postprocessing

**Fig. 2:**
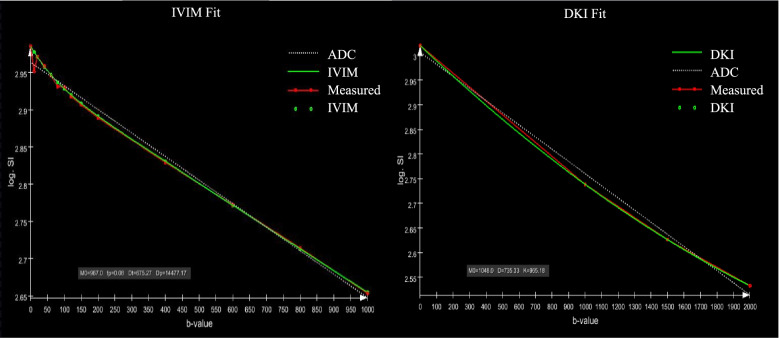
Fitted plots of IVIM and DKI

**Fig. 3:**
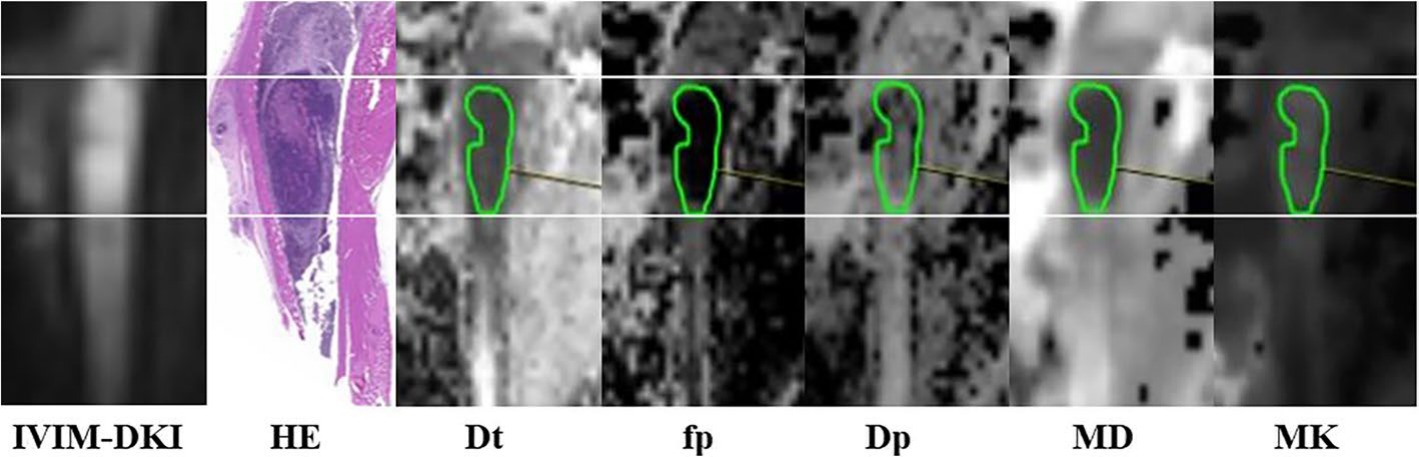
Figure shows HE staining of tumor specimen and corresponding IVIM-DKI image and the derived parameters maps

Image analysis was performed using the Body Diffusion Toolbox (Siemens Healthcare GmbH, Erlangen, Germany) on the Siemens workstation (syngo.via). Two experienced radiologists analyzed the images independently, with any disagreements being resolved through consultation. It should be noticed that IVIM and DKI have different b-values selected for subsequent processing. The IVIM was fitted on the basis of images with b-values of 0, 10, 20, 30, 40, 50, 80, 100, 150, 200, 400, 600, 800, and 1000 s/mm^2^. In addition, DKI was fitted according to images with b-values of 0, 1000, 1500, and 2000 s/mm^2^. The fitting diagram was attached to Fig. 2. Considering the heterogeneity of the tumor, the radiologist manually defined the region of interest (ROI) of the tumor contour on the corresponding tissue diffusion (Dt) map of the highest b-value, based on IVIM-DKI images of the largest sagittal plane of the tumor, using the same level of HE-stained specimens as a guide (Fig. 3). The entire solid tumor volume is covered, carefully avoiding cystic, hemorrhagic and artifact areas, and plotted three times to obtain a final average. The ROI was then automatically replicated to other parameter maps, including pseudo-diffusion (Dp), perfusion fraction (fp) for IVIM, and mean diffusion coefficient (MD) and mean kurtosis (MK) for DKI.

### Statistical analysis

All statistical analyses were performed using SPSS (version 22.0; Chicago, IL, USA) and MedCalc (version 19.0.7; Ostend, Belgium). The Kolmogorov–Smirnov test of normality was performed for analyzing normality at *p* value>0.05. The mean value of the preprocessing parameters and the early changes after 5 days of radiotherapy were compared between the pre-treatment and post-treatment group using independent sample *t*-tests. A one-way ANOVA and Tukey post hoc multiple comparisons were used to analyze the differences in each quantitative diffusion parameters between the pre-treatment, considerable effect, and slight effect malignant bone tumor groups. Correlations between the diffusion parameters of the IVIM and DKI models were analyzed using Pearson correlation. Binary logistic regression analysis was performed to establish a diagnostic model with a combination of different parameters including Dt (cellularity), fp (vascularity), and MK (microstructural complexity) of IVIM and DKI, according to the results of the analyses stated above. Receiver operating characteristics (ROC) curves were created and the areas under the curves (AUCs) were calculated to evaluate the diagnostic performance of each diffusion parameter and their combinations for differentiating between considerable effect and slight effect groups. A *p* value of < 0.05 was considered significant for the above analyses.

## Results

**Fig. 4:**
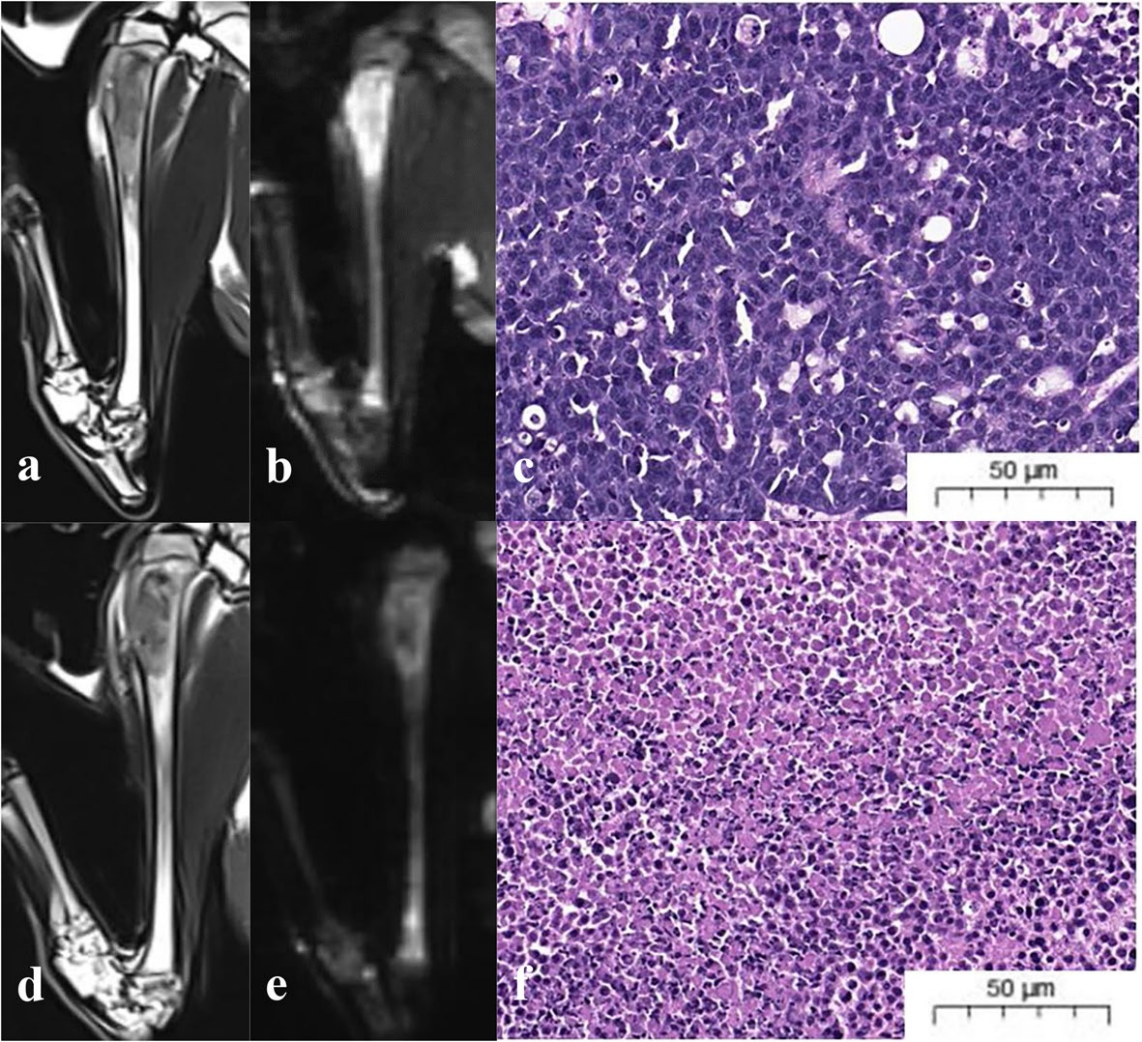
IVIM-DKI and histopathological images of rabbit VX2 malignant bone tumor models. The images are the T2FS，IVIM-DKI and the corresponding HE images (200×) before (**a**, **b**, **c**) and after (**d**, **e**, **f**) radiotherapy. Before radiotherapy, the tumor cells were dense and distributed in clusters. After treatment, cytoplasmic necrosis increased and tumor cell density decreased significantly

Solid intramedullary tumor transplantations were successfully established in the right tibial tubercle in 100% (*n* = 50) of the rabbits, of which two died spontaneously and one died after radiotherapy. Of the 47 lesions in the remaining rabbits, 15 were in the pre-treatment group, 18 were in the considerable effect group, and 14 were in the slight effect group. The tumors were found to be represented by elliptic and irregular osteolytic bone destruction. The tumors showed low signal intensity on T2FS and inhomogeneous high signal on IVIM-DKI. The borders of some lesions became clear after radiotherapy, with the IVIM-DKI signal in the solid area of the tumor decreasing, and the range of the central necrotic area increasing (Fig. 4).

**Fig. 5:**
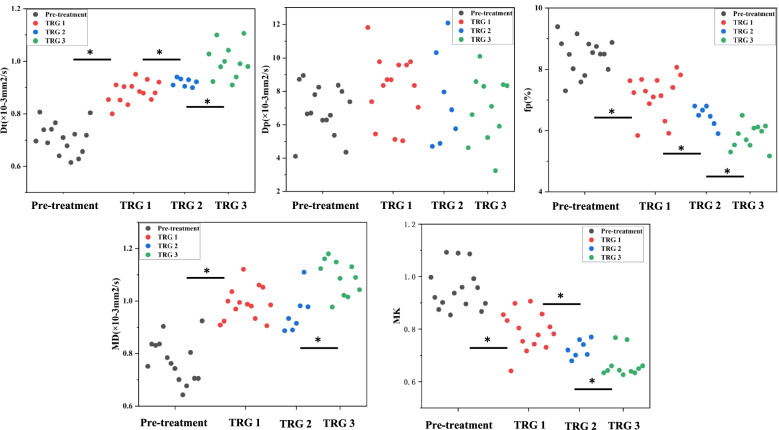
Dot plots show the results of IVIM and DKI parameters analysis in the pre-treatment, TRG1, TRG2, TRG3. ∗ *p* < 0.05. TRG, tumor regression grade

After treatment, the D_t_, and MD values showed an upward trend (both *p* <  0.001), while the f_p_ and MK values showed a decreasing trend (both *p* <  0.001). The distribution of IVIM-DKI quantitative parameters distinguishing the grades of tumor regression is shown in Fig. 5, and the results of comparisons of the IVIM-DKI quantitative parameters and tumor cell counts between the pre-treatment, considerable effect, and slight effect groups are listed in Table 1. All quantitative parameters except Dp showed a significant difference between the considerable effect and slight effect groups (*p* <  0.05).

**Table 1:** Summary of Diffusion Parameters in each group

Variable	Pre-treatment	Post-treatment		P1	P2	P3
		Considerable effect	Slight effect			
Dt(× 10^− 3^ mm^2^/s)	0.71 ± 0.06	0.95 ± 0.06	0.88 ± 0.04	< 0.001	< 0.001	0.007
fp(%)	8.44 ± 0.59	6.51 ± 0.77	7.14 ± 0.69	< 0.001	< 0.001	0.036
Dp(×10^− 3^ mm^2^/s)	6.91 ± 1.50	7.17 ± 2.31	8.19 ± 1.99	0.929	0.207	0.330
MK	0.95 ± 0.08	0.73 ± 0.06	0.79 ± 0.07	< 0.001	< 0.001	0.031
MD(×10^−3^ mm^2^/s)	0.77 ± 0.08	1.04 ± 0.10	0.99 ± 0.06	< 0.001	< 0.001	0.042
TCD (%)	43.55 ± 4.86	19.15 ± 3.40	33.58 ± 11.10	< 0.001	< 0.001	< 0.001

P1: Pre-treatment vs. Considerable effect, P2: Pre-treatment vs. Slight effect, P3: Considerable effect vs. Slight effect

*D*_*t*_ Tissue diffusion, *f*_*p*_ Perfusion fraction, *D*_*p*_ Pseudo-diffusion, *MK* Mean kurtosis, *MD* Mean diffusion coefficient, *TCD* Tumor cell density

**Fig. 6:**
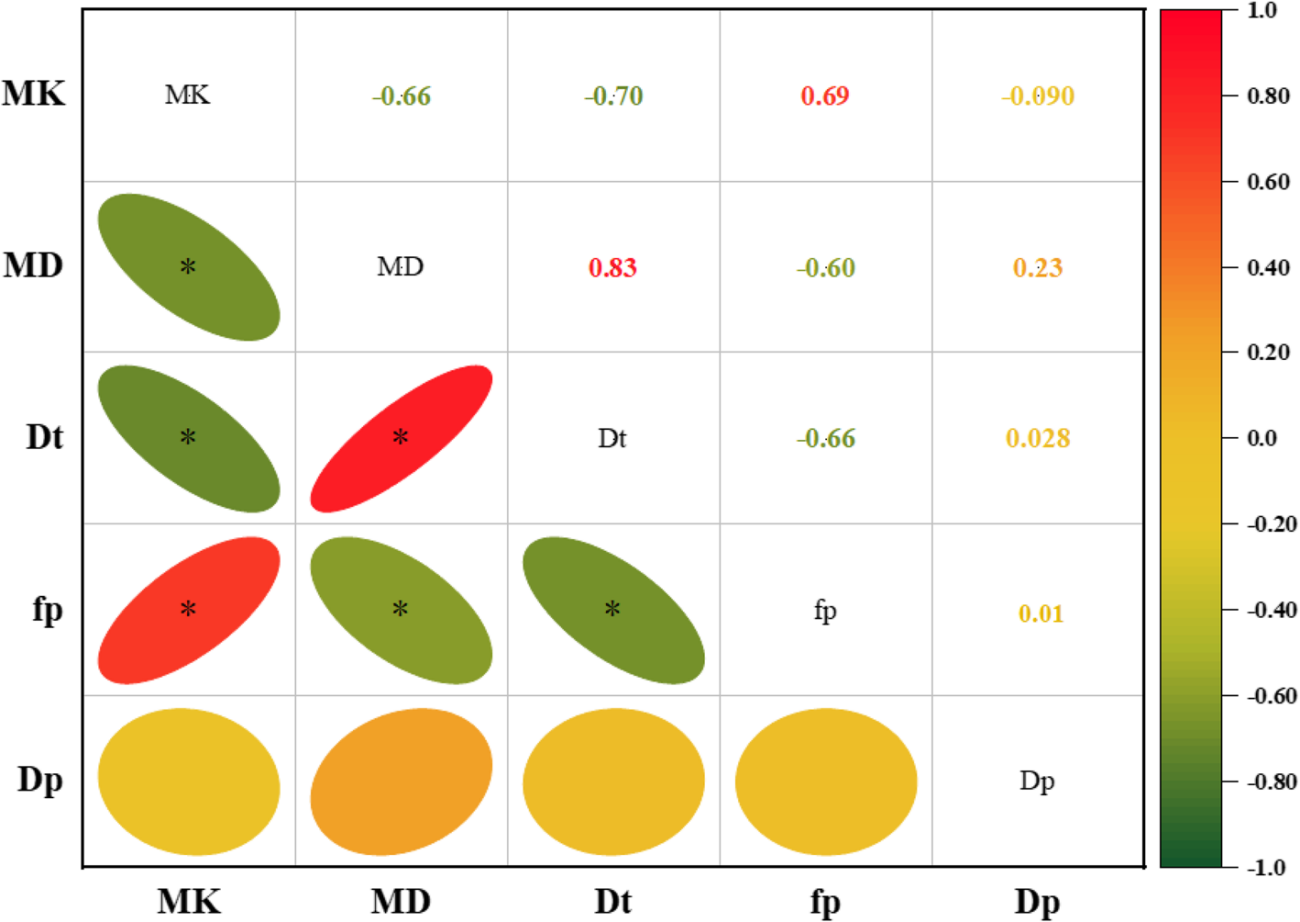
Heat map depicting the Pearson correlation between IVIM-DKI parameters. The numbers represent the Pearson coefficient. ∗ *p* < 0.05

Figure 6 summarizes the correlations between the dispersion parameters originating from IVIM and those from DKI. D_t_ and MD values showed a negative correlation with MK value (r_Dt&MK_ = -0.701, *p* <  0.001, r_MD &MK_ = -0.662, *p* <  0.001), and showed a negative correlation with f_p_ value (r_Dt&fp_ = − 0.660, *p* < 0.001, r_MD &fp_ = − 0.604, *p* < 0.001). Futhermore, MK was positively correlated with f_p_ (r_MK&fp_ = 0.690, *p* < 0.001). None of the other IVIM or DKI parameters showed any significant correlation with D_p_ (*p* > 0.05).

**Fig. 7:**
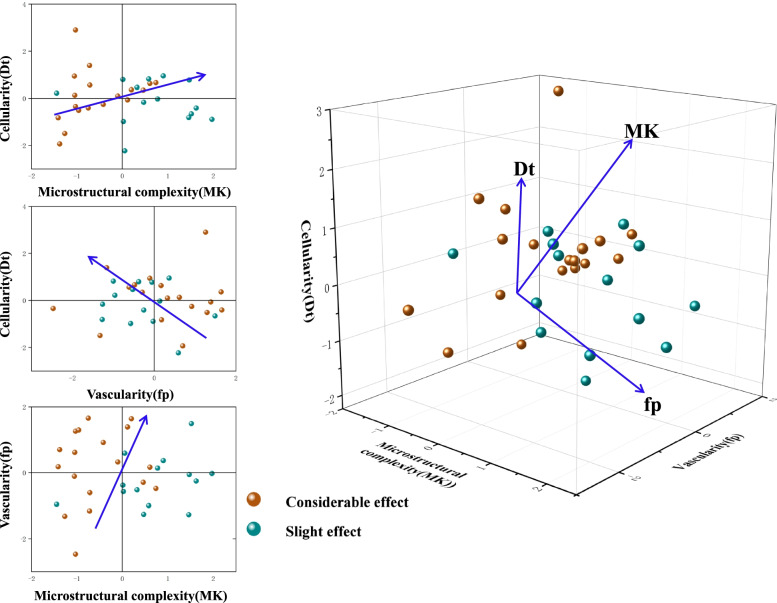
Discriminating the considerable and slight effect of rabbits with malignant bone tumor model after radiotherapy in 2D and 3D data spaces constructed from a combination of different biological information. Each point in the graph represents each animal in the considerable and slight effect group

**Fig. 8:**
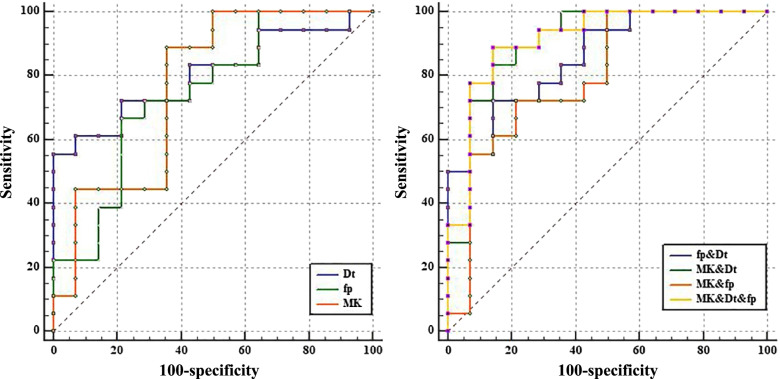
The result of ROC curve analysis to depict the efficacy of various parameters and their combinations for predicting the response outcomes of VX2 malignant bone tumor treatment

A binary logistic regression model including the three variables Dt, MK, and fp was established. Combination of different biological information from IVIM-DKI derived parameters was used to construct two-dimensional (Dt and MK, Dt and fp, fp and MK) and three-dimensional data spaces to discriminate between considerable and slight effect groups after radiotherapy for rabbits with bone tumor model (Fig. 7). The results showed that the differentiation performance was superior to that of individual parameters by combining the second derived parameters. Table 2 describes the diagnostic performance of the different parameters and their combinations (Fig. 8). The AUCs of the D_t_, MK, and f_p_ values were 0.802, 0.762, and 0.742, respectively. The best results for differentiating considerable effect from slight effect after treatment were obtained when all three IVIM-DKI-derived parameters were combined (AUC = 0.913).

**Table 2:** Diagnostic performance for IVIM-DKI indexes and their combinations in assessing radiotherapeutic response

Variable	Youden Index	AUC	(95%CI)	Sensitivity (%)	Specificity (%)
Dt	0.556	0.802	(0.623–0.921)	55.56	100.00
fp	0.452	0.742	(0.558–0.880)	66.67	78.57
MK	0.532	0.762	(0.579–0.894)	88.89	64.29
Dt and fp	0.579	0.857	(0.688–0.955)	72.22	85.71
Dt and MK	0.691	0.905	(0.748–0.980)	83.33	85.71
fp and MK	0.508	0.798	(0.619–0.918)	72.22	78.57
Dt and fp and MK	0.746	0.913	(0.758–0.983)	88.89	85.71

*Dt* Tissue diffusion, *fp* Perfusion fraction, *Dp* Pseudo-diffusion, *MK* Mean kurtosis, *MD* Mean diffusion coefficient

## Discussion

In this study, we created a model combining IVIM and DKI parameters to predict changes in tumor biology after radiotherapy in rabbits with malignant bone tumor model. The results showed that the functional parameters of IVIM and DKI were statistically correlated with the radiotherapy response of the malignant bone tumor model. After treatment, the D_t_ and MD values all showed a raising trend, while the fp and MK values showed a downward trend, with statistically significant differences between considerable effect and slight effect groups (*p* = 0.007, 0.036, 0.042, and 0.031, respectively). The logistic regression model combining the IVIM- and DKI-derived parameters of Dt, fp, and MK, which reflected biological information, yielded an optimum AUC of 0.913. In addition, we showed a close relationship between IVIM and DKI-derived parameters reflecting vascularity, cellularity, and microstructural complexity, which further demonstrates that combining these technologies can improve the accuracy of therapy response assessment.

The individual parameters derived from IVIM and DKI, except for Dp, showed potential for monitoring the efficacy of radiotherapy for malignant bone tumors. Radiotherapy works through the intrinsic pathway involving the permeability of the outer mitochondrial membrane, which leads to morphological changes and DNA fragmentation. In the process of tumor cell apoptosis, the extracellular space is increased and the diffusion of water molecules in the tumor infiltration area is promoted [18, 19]. Therefore, the Dt and MD values increased in the infiltration zone after radiotherapy. Additionally, as tumor cell density and nuclear atypia decrease after treatment, the degree to which dispersed water molecules deviate from a Gaussian distribution is reduced, which can be quantified by a reduction in MK values. Compared with the considerable effect group, the MK values of the slight effect group were slightly higher. The reason for this result may be that the tumor cells in the slight effect group faced higher hypoxia and an acidic environment, which may have led to necrosis and fibrosis. Corresponding changes increased the complexity of the tissue structure, making it insensitive to radiotherapy and reducing the therapeutic effect [20]. The reason for the decrease in fp values after radiotherapy is that the tumor microvascular system is severely damaged, along with macroscopic manifestations such as intratumoral hemorrhage, resulting in a decrease in the microcirculation perfusion fraction [21].

In comparison with previous studies that directly combined multiple DWI models, our study focused on evaluating the potential clinical value of different parameters calculated from a combined IVIM-DKI model for determining the response to radiotherapy of malignant bone tumors. The results of the binary logistic regression and ROC analysis demonstrated that for predicting the efficacy of radiotherapy for malignant bone tumors the IVIM-derived Dt value that reflects cellularity was the most sensitive parameter, followed by the DKI-derived MK that reflects microstructural complexity, and the IVIM-derived fp that reflects vascularity. Some previous studies showed similar results. Noriyuki et al. demonstrated that the percentage change in Dt value showed high diagnostic performance for evaluating treatment response in patients with squamous cell carcinoma of nasal or sinonasal regions [22]. Zhang et al. suggested that the IVIM-derived Dt provided higher value for predicting the chemotherapeutic response of colorectal liver metastases than parameters derived from DKI [23]. However, differing from our results, Zhong et al. found that, compared with other parameters, the MK value was the most valuable surrogate biomarker for non-invasively evaluating the characteristics of nasopharyngeal carcinoma tissue [24]. Similarly, Xu et al. found that MK was most effective in glioma grading [25]. We believe that the above inconsistency can be explained by the following points. First, there are substantial physiological, pathological, and biological differences between different types of diseases and specific pathological processes. The VX2 tumor cells selected in this study have similar biological behaviors to human malignant bone tumors [26]. Cells in the division phase are sensitive to radiotherapy, resulting in a decrease in tumor cell density and an increase in intercellular space after treatment. As the b-value increases, bone tumors may show faster signal attenuation than other tissues. Second, the b-values selected enabled Dt to be calculated without the influence of perfusion, allowing better characterization of the true diffusion limitations caused by cell changes. Finally, data analysis may also lead to differences in conclusions. For example, the combined IVIM-DKI data differs from analysis using DKI and IVIM parameters separately [27].

The cellularity-related parameters Dt and MD and the microstructure complexity-related parameter MK were significantly negatively correlated, as was the vascularity-related parameter fp, whereas MK and fp were significantly positively correlated. The reason for these results may be the abundant microvessels in the solid area of the tumor, with many immature blood vessels and high permeability. Radiotherapy blocked the growth of tumor neovascularization, resulting in a decrease in tumor microvessels, blood perfusion, and tissue necrosis, making the tumor more hypoxic and reducing the density of cells [19]. In summary, the correlation of cellularity-, vascularity-, and microstructural complexity-reflecting parameters derived from the IVIM-DKI model indicated the necessity to integrate these measurements to obtain more comprehensive tumor characteristics. In the ROC analysis, among all the parameters and their different combinations, the diagnostic efficacy for predicting radiotherapy outcomes for malignant bone tumors was highest when the biological information represented by the above three IVIM-DKI parameters was integrated.

Our study is subject to some limitations that must be highlighted. First, we monitored the early treatment response to radiotherapy, rather than evaluating dynamic changes over long-term treatment. Therefore, a longer observation period is warranted to refine the above results. Second, this study focused on evaluating the histological characteristics of animal model, which may not accurately reflect the conditions of human malignant bone tumors, and further clinical investigations are necessary. Furthermore, the ROIs used were outlined on a single image slice, and we will outline the whole tumor in the future to explore the impact on the assessment results. Finally, logistic regression was applied to conjoint analysis of IVIM and DKI. In future research, relevant clinical information and other complex statistical models should also be introduced.

## Conclusions

In conclusion, this study combined IVIM and DKI methods to non-invasively obtain parameters reflecting cellular, vascular, and microstructural changes in malignant bone tumors after radiotherapy, which improved monitoring of the efficacy of tumor radiotherapy. Furthermore, because of the tight correlations among the derived parameters, the integration of different parameters holds great potential for achieving more comprehensive tumor characterization before morphological size change occurs, which will have guiding significance for effective individualized treatment.

## Supplementary Information

**Additional file 1: Supplemental Fig. 1.** Chemical and physical design of quantitative IVIM-DKI phantom. **Supplemental Fig. 2.** Bland–Altman plots of differences between different concentrations measurements are shown, further illustrating high similarity in IVIM-DKI parameters quantification between test and retest group. **Supplemental Table 1.** The coefficient of variation (CV) Values for Phantom Test-Retest Acquisitions.

## Data Availability

The data used and/or analyzed in this study were obtained from experiments.

## Abbreviations

DWI: Diffusion-weighted imaging
ADC: Apparent diffusion coefficient
IVIM: Intravoxel incoherent motion
DKI: Diffusion kurtosis imaging
FS: Fat-saturated
FSE: Fast spin-echo
HE: Hematoxylin-eosin
ROI: Regions of interest
Dt: Tissue diffusion
Dp: Pseudo-diffusion
fp: Perfusion fraction
MD: Mean diffusion coefficient
MK: Mean kurtosis
ROC: Receiver operating characteristic
AUCs: Areas under the curves
