# Supplementary material for: Intravoxel incoherent motion imaging combined with diffusion kurtosis imaging to assess the response to radiotherapy in a rabbit VX2 malignant bone tumor model
Source: Cancer Imaging. 2022 Sep 5;22:47. doi: 10.1186/s40644-022-00488-w (PMC9446876; doi:10.1186/s40644-022-00488-w)

Supplemental Material


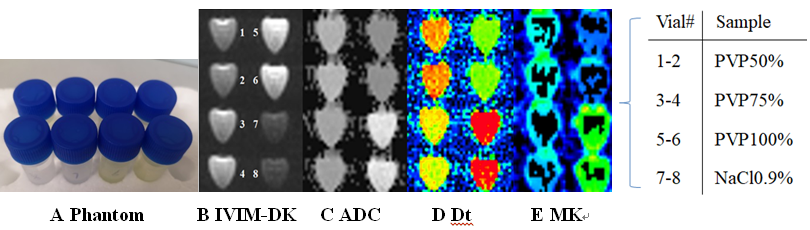
Polyvinylpirrolidone (PVP) solutions in water at 50%, 75%, and 100%, as well as NaCl solutions at 0.9%

Supplemental Figure 1: Chemical and physical design of quantitative IVIM-DKI phantom.


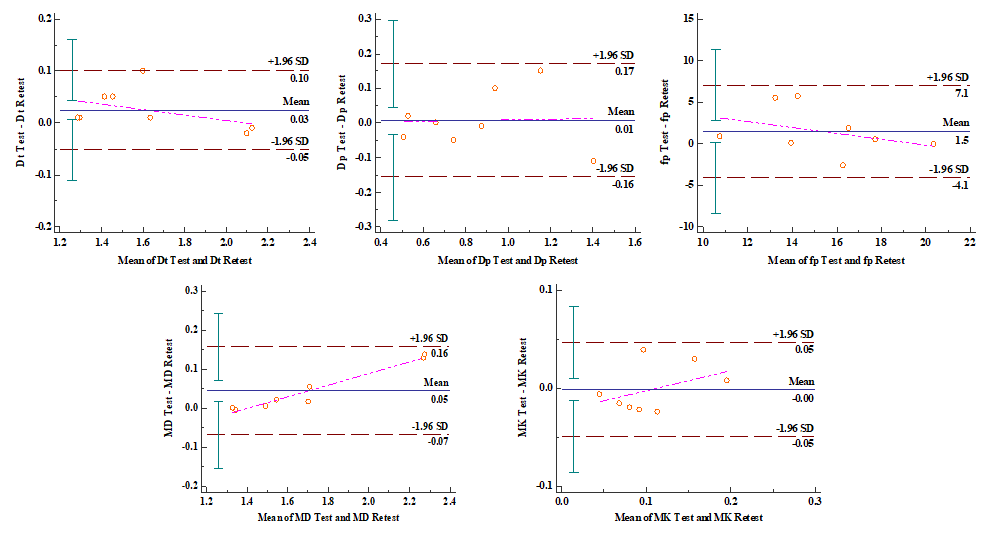
Supplemental Figure 2: Bland–Altman plots of differences between different concentrations measurements are shown, further illustrating high similarity in IVIM-DKI parameters quantification between test and retest group

Supplemental Table 1: The coefficient of variation (CV) Values for Phantom Test-Retest Acquisitions


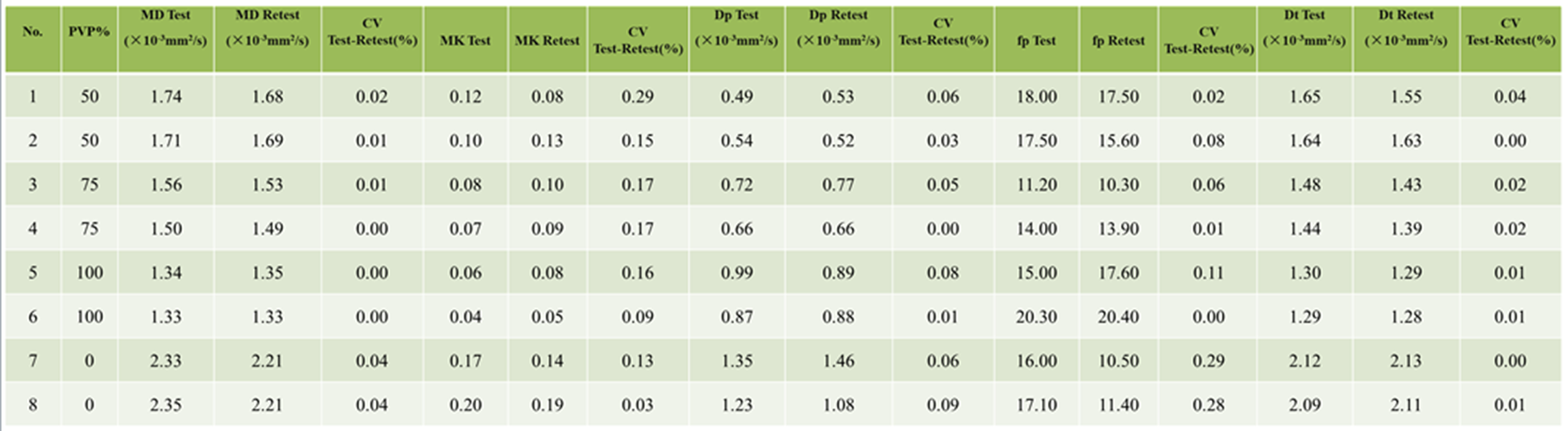

Supplement: Supplementary file 1 — Additional file 1: Supplemental Fig. 1. Chemical and physical design of quantitative IVIM-DKI phantom. Supplemental Fig. 2. Bland–Altman plots of differences between different concentrations measurements are shown, further illustrating high similarity in IVIM-DKI parameters quantification between test and retest group. Supplemental Table 1. The coefficient of variation (CV) Values for Phantom Test-Retest Acquisitions. [file 40644_2022_488_MOESM1_ESM.docx]
